# Variety of Bacterial Pathogens in Ticks Removed from Humans, Northeastern China

**DOI:** 10.3390/microorganisms13122862

**Published:** 2025-12-16

**Authors:** Xiao-Ling Su, Jin-Ling Ye, Ming-Zhu Zhang, Yi-Fei Wang, Yi Sun, Ya-Fei Wu, Cai Bian, Nan-Nan Yao, Yuan-Chun Zheng, Jia-Fu Jiang, Xiao-Min Zheng, Wu-Chun Cao

**Affiliations:** 1 State Key Laboratory of Pathogen and Biosecurity, Academy of Military Medical Sciences, Beijing 100071, China; suxll@outlook.com (X.-L.S.); sunyi7310@sina.com (Y.S.); 15810174122@139.com (J.-F.J.); 2 Mudanjiang Forestry Central Hospital, Mudanjiang 157011, China; yejinling1986@126.com (J.-L.Y.); 13604637331@163.com (C.B.); 18545306345@163.com (N.-N.Y.); zhengyuanchun888@126.com (Y.-C.Z.); 3 Institute of EcoHealth, School of Public Health, Shandong University, Jinan 250012, China; 18216013722@163.com (M.-Z.Z.); wangyf419@163.com (Y.-F.W.); 4 National Institute of Pathogen Biology, Chinese Academy of Medical Sciences & Peking Union Medical College, Beijing 102629, China; wuyafei6711@163.com

**Keywords:** *Borreliella*, *Borrelia*, *Ehrlichia*, *Rickettsia*, molecular detection, molecular epidemiology study, tick-borne pathogens, ticks

## Abstract

Ticks transmit diverse pathogens, posing significant public health threats in northeastern China; clarifying human-biting tick species and their carried pathogens is crucial for risk assessment and prevention. This study was designed as a pathogen detection and molecular epidemiological investigation. During the May-August period of each of 2023 and 2024, 232 ticks that had bitten humans were collected from a local sentinel hospital. Ticks were morphologically identified, followed by DNA extraction, semi-nested/nested PCR targeting specific genetic markers, and Sanger sequencing to detect bacterial pathogens. Four tick species were found, with *Ixodes persulcatus* dominant (87.9%), followed by *Dermacentor silvarum* (6.9%), *Haemaphysalis concinna* (3.5%), and *Haemaphysalis japonica* (1.7%). Seven bacterial pathogens were detected, including *Candidatus* Rickettsia tarasevichiae (35.3%) and *Borreliella garinii* (17.2%); 49.6% of ticks were pathogen-positive. The coinfection rate was 13.8%, most commonly *Candidatus* R. tarasevichiae plus *B. garinii*, and *I. persulcatus* carried all pathogens. These findings provide basic data on human-biting tick species and their bacterial pathogen spectrum in the region, serving as a reference for subsequent tick-borne disease research and targeted prevention.

## 1. Introduction

Ticks are arthropods that transmit various pathogens to humans, second only to mosquitoes in spreading infectious diseases worldwide [1]. Tick-borne infectious diseases have been continuously emerging in mainland China, particularly over the past decade, and are becoming an increasing public health threat [2,3]. A wide range of tick-borne pathogens are responsible for these emerging diseases, involving dozens of tick species that are widely distributed across China, suggesting that cases of emerging tick-borne diseases may be underdiagnosed [3]. Detecting pathogens carried by ticks while they are actively feeding on humans provides an effective approach to actively identify and assess the risk of tick-borne diseases, and also serves as an important basis for optimizing prevention and response strategies [4].

Mudanjiang Forestry Central Hospital is situated in Mudanjiang City (45° N, 129° E), southeastern Heilongjiang Province, Northeast China, within the Changbai Mountains and adjacent to the Russian border. The area lies in the mid-north-temperate zone and has a humid continental monsoon climate with warm, rainy summers; the high forest coverage and abundant moisture provide optimal temperature and humidity conditions for the survival, activity and reproduction of *Ixodes persulcatus* and other tick species. The municipality has 2.8 million inhabitants, most of whom are engaged in forestry, agriculture or outdoor recreation—occupations that ensure frequent contact with tick habitats and increase the risk of tick bites [5]. As one of the largest hospitals in the forest areas of northeastern China and a designated sentinel site for tick-borne disease surveillance, Mudanjiang Forestry Central Hospital plays a key epidemiological role in this ecologically relevant region [6].

As a key sentinel facility in northeastern China, several emerging tick-borne diseases caused by pathogens have been identified there. *Candidatus* Rickettsia tarasevichiae, which was first identified in *I. persulcatus* in Russia and was identified in 2013 as the inaugural human cases in China at this hospital; patients presented with fever, headache and an eschar at the bite site, confirming trans-border transmission [7]. In the same year, *Rickettsia sibirica* subsp. *sibirica* BJ-90, an agent of North Asian spotted fever and previously reported in Mongolia, Siberia, and northeastern China, was identified, capable of causing high fever, rash and multi-organ injury [8]. In 2014, *Rickettsia conorii* subsp. *raoultii* was detected for the first time in northeastern China, both in the blood of a tick-bitten patient and in local *Dermacentor silvarum* ticks, indicating the establishment of a natural focus in the region [9]. *Anaplasma capra* was identified in 2015 via metagenomic sequencing from the blood of a febrile patient with thrombocytopenia in Mudanjiang, expanding the spectrum of zoonotic anaplasmosis [5]. *Neoehrlichia mikurensis*, first reported in Europe in 2009, was detected in 2012 in the blood of an immunocompetent patient at the hospital, representing the first Chinese case and extending the geographic range of this vasculotropic pathogen [10]. Yezo virus and Xue-Cheng virus, two novel members of the genus Orthonairovirus, were isolated from the blood of febrile patients and associated with acute fever and cytopenia [6,11]. Collectively, these findings highlight the high diversity and active circulation of tick-borne pathogens in the Mudanjiang forest region, providing a robust scientific basis for continuous surveillance and control.

In addition to patients presenting with fever following tick bites, local residents also visit the outpatient department carrying blood-feeding ticks still attached to their bodies, and medical staff assist in removing the ticks. There is a lack of systematic research on the species of ticks that bite humans and the pathogens they carry. Therefore, this study intends to identify the species of human-derived ticks collected from the hospital, and detect the bacterial pathogens they carry. The aims were to investigate the species composition of human-biting ticks in Northeast China, determine the infection spectrum and prevalence of tick-borne pathogens, and serve as an important reference for subsequent studies, including the analysis of epidemiological characteristics of tick-borne diseases, the construction of risk assessment models, and the optimization of targeted prevention and control strategies.

## 2. Materials and Methods

### 2.1. Sample Collection

Tick specimens were obtained from Mudanjiang Forestry Central Hospital, Heilongjiang Province, during the May–August period of each of 2023 and 2024. The study protocol was approved by the Ethics Committee of Mudanjiang Forestry Central Hospital. All participants provided informed consent for the use of their anonymized tick samples. Ticks were submitted by patients who presented at the hospital with attached ticks, which were carefully removed from the skin using fine forceps by medical staff, placed into sterile 1.5 mL microcentrifuge tubes. Ticks with intact bodies were collected for this study, and stored at –80 °C until further processing. All specimens were morphologically identified and classified by an entomologist (Y.S.) prior to molecular processing, using standard taxonomic keys [12,13,14,15,16].

### 2.2. DNA Extraction

Prior to nucleic acid extraction, ticks were surface-sterilized with 75% ethanol for 10 min, rinsed twice with sterile nuclease-free water, and air-dried on sterile filter paper. Each tick was then transferred into a 1.5 mL microcentrifuge tube containing 2–3 sterile steel beads and 200 μL RNase-free water. Homogenization was performed using a tissue grinder under the following conditions: 4 °C, 55 Hz, 300 s (Wanbai Biotechnology Co., Ltd., Suzhou, Jiangsu, China). DNA was subsequently extracted from the homogenates using the AllPrep DNA/RNA Mini Kit (Qiagen, Valencia, CA, USA) according to the manufacturer’s instructions. The nucleic acids were eluted in 100 μL RNase-free water for downstream analyses. All nucleic acid extraction procedures were performed in a biological safety cabinet to maintain sterility and prevent contamination. Following extraction, DNA samples were stored at −80 °C until PCR assays.

### 2.3. PCR Assays and Sequencing

Pathogen detection was performed by PCR (semi-nested and nested PCR) amplification of specific genetic fragments: the outer membrane protein A (*ompA*), citrate synthase (*gltA*), and 17 kDa surface antigen (*17 kDa*) genes of spotted fever group *Rickettsia*; the *gltA*, heat shock protein (*groEL*), and 16S rRNA genes of *Ehrlichia*; the 16S rRNA and *groEL* genes of *N. mikurensis*; the 16S rRNA and 5S-23S rRNA gene for *Borrelia* (Table S1). PCR reaction mixtures were prepared on a dedicated clean bench to minimize aerosol contamination. Amplification reactions were conducted using PCR instruments from Applied Biosystems (Thermo Fisher Scientific, Waltham, MA, USA). The DNA of homogenized ticks was used as a positive control, whereas ddH_2_O was set as the negative control. PCR amplicons were subjected to paired-end Sanger sequencing by Beijing Tianyi Huiyuan Biotechnology Co., Ltd. (Beijing, China). Raw sequences were proofread, edited, and assembled using CLC Main Workbench (v5.0) for subsequent analyses. All pathogen gene sequences obtained in this study have been deposited in GenBank (Table S2).

### 2.4. Phylogenetic Analysis

Sequences obtained from PCR products were initially queried using BLAST (NCBI; http://www.ncbi.nlm.nih.gov/BLAST, accessed on 20 January 2025) and aligned with reference sequences from GenBank using MAFFT (v7.487) [17]. Phylogenetic trees were reconstructed using the maximum likelihood (ML) method implemented in IQ-TREE (v2.2.0.3) with 1000 bootstrap replicates [18]. Tree annotation and visualization were performed with iTOL (v7.1) (https://itol.embl.de, accessed on 11 March 2025) [19]. Genetic distances were calculated using MEGA 11 [20].

### 2.5. Statistical Analyses

Proportions and corresponding 95% confidence intervals (95% CI) were estimated using the Wilson score method, which provides robust interval estimates, particularly for small sample sizes or extreme proportions. Differences in positivity rates between groups were assessed using the Chi-square (χ^2^) test [21]. Statistical analyses were conducted using R software (v4.4.2), and a two-tailed *p* value < 0.05 was considered statistically significant.

## 3. Results

### 3.1. Tick Sampling

A total of 232 fed ticks were collected from patients at the sentinel hospital during May–August in 2023 and 2024. Morphological inspection showed that all 232 ticks were adult females at the semi-engorged stage. *I. persulcatus* was the predominant species (204/232, 87.9%; 95% CI: 83.1–91.5%), followed by *D. silvarum* (16/232, 6.9%; 95% CI: 4.3–10.9%), *Haemaphysalis concinna* (8/232, 3.5%; 95% CI: 1.8–6.7%), and *Haemaphysalis japonica* (4/232, 1.7%; 95% CI: 0.7–4.4%).

### 3.2. Phylogenetic Analysis of Different Tick-Borne Pathogens

**Figure 1:**
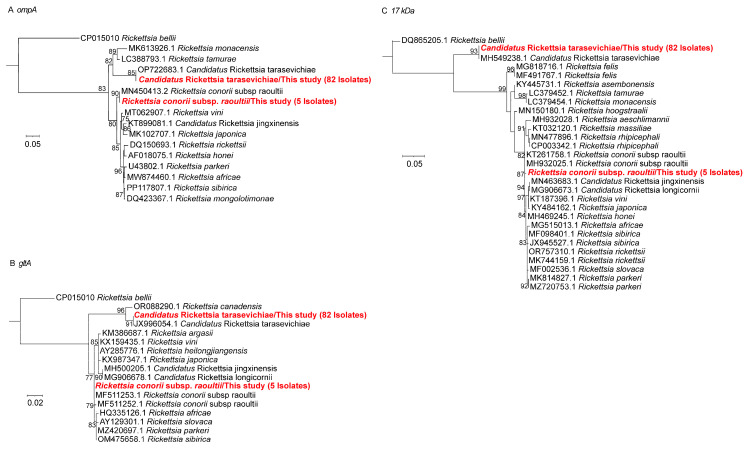
Phylogenetic trees of *Rickettsia* were constructed using the maximum likelihood (ML) method with 1000 bootstrap replicates. (**A**) *ompA* gene; (**B**) *gltA* gene; (**C**) *17 kDa* gene. The evolutionary positions of samples that tested positive for fragments are highlighted in red text.

Nested PCR targeting the *ompA*, *gltA*, and *17 kDa* genes detected 87 positive samples, all of which yielded consistent results across the three loci. Sequence homology and phylogenetic analyses revealed two *Rickettsia* species: *Candidatus* R. tarasevichiae and *R. conorii* subsp. *raoultii*. Eighty-two samples were identified as *Candidatus* R. tarasevichiae, with *ompA* sequences showing 100% identity to strains from *I. persulcatus* in Russia (GenBank accession no. OP722683.1). The *gltA* sequences shared 99.7–100% similarity with *R. tarasevichiae* detected in human samples from Heilongjiang, China (GenBank accession no. JX996054.1), while *17 kDa* sequences showed 100% identity with human-derived *Candidatus* R. tarasevichiae from China (GenBank accession no. MH549238.1). The remaining five samples were identified as *R. conorii* subsp. *raoultii*, with *ompA*, *gltA*, and *17 kDa* sequences all showing 100% identity to sequences previously reported in *Haemaphysalis longicornis*, *I. persulcatus*, and *Dermacentor nuttalli* from China (GenBank accession nos. MN450413.2, MF511253.1, and MH932025.1, respectively) (Figure 1).

**Figure 2:**
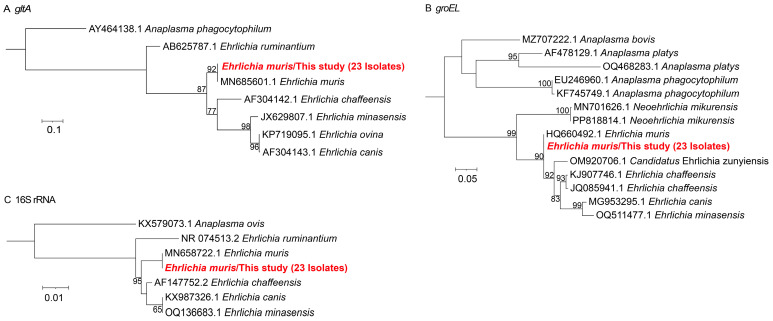
Phylogenetic trees of *E. muris* were constructed using the maximum likelihood (ML) method with 1000 bootstrap replicates. (**A**) *gltA* gene; (**B**) *groEL* gene; (**C**) 16S rRNA gene. The evolutionary positions of samples that tested positive fragments are highlighted in red text.

A total of 23 samples tested positive for *Ehrlichia* based on the *gltA*, *groEL* and16S rRNA. Both *gltA* and 16S rRNA sequences clustered with *E. muris* detected in *Myodes rufocanus* from Russia (GenBank accession nos. MN685601.1 and MN658722.1) with 100% identity, while *groEL* sequences showed 100% identity to *E. muris* from *Ixodes dammini* in the United States (GenBank accession no. HQ660492.1) (Figure 2).

**Figure 3:**
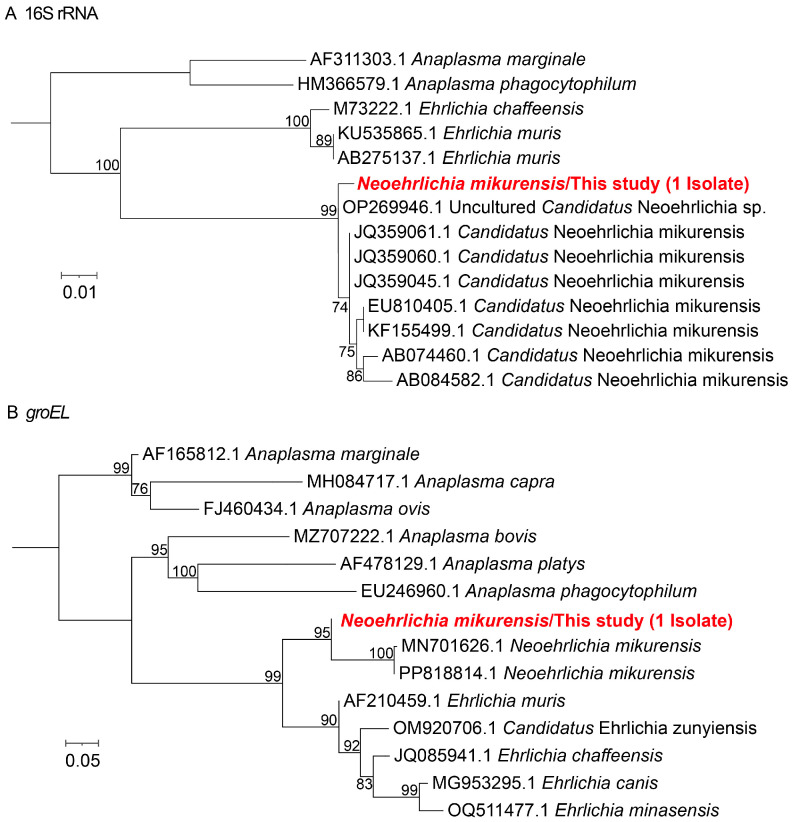
Phylogenetic trees of *N. mikurensis* were constructed using the maximum likelihood (ML) method with 1000 bootstrap replicates. (**A**) 16S rRNA gene; (**B**) *groEL* gene. The branch for the positive sample is highlighted in red text.

In addition, one sample was positive for *N. mikurensis*, with 16S rRNA sequence sharing 99.7% similarity with uncultured *Candidatus* Neoehrlichia detected in *Ixodes ricinus* from Italy (GenBank accession no. OP269946.1), and *groEL* sequence showing 93.1% similarity to *N. mikurensis* detected in rodent kidneys from China (GenBank accession no. PP818814.1) (Figure 3).

**Figure 4:**
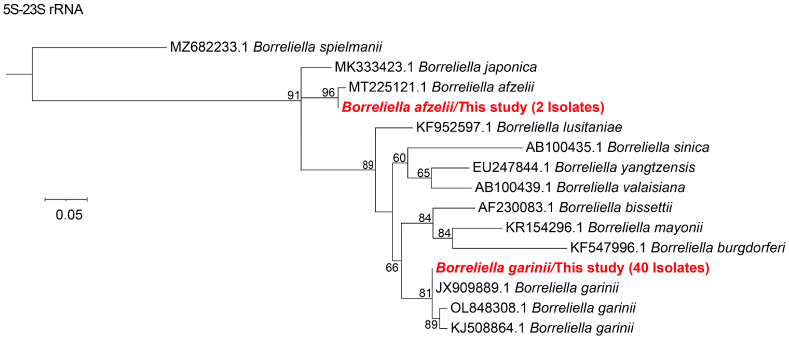
The phylogenetic tree of 5S-23S rRNA gene of *Borrelia burgdorfer* s.l. was constructed using the maximum likelihood (ML) method with 1000 bootstrap replicates. The evolutionary positions of samples that tested positive fragments are highlighted in red text.

**Figure 5:**
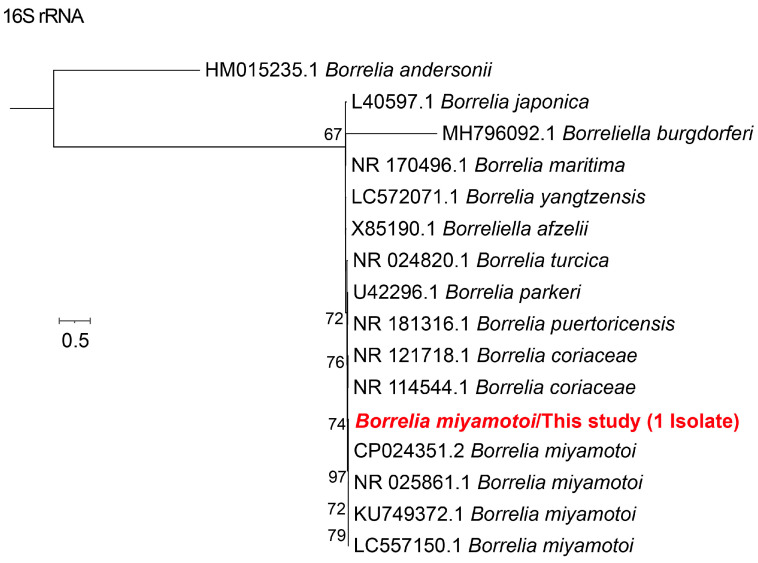
The phylogenetic tree of 16S rRNA gene of *B. miyamotoi* was constructed using the maximum likelihood (ML) method with 1000 bootstrap replicates. The branch for the positive sample is highlighted in red text.

Based on sequence similarity and phylogenetic analyses of the 5S-23S rRNA, two *Borreliella* species were identified: *B. garinii* and *B. afzelii*. Forty samples were positive for *B. garinii*, with 5S-23S rRNA sequences sharing 95–100% nucleotide identity with strains isolates detected in *I. ricinus* from the Baltic Sea: Aland Islands (GenBank accession no. JX909889.1). Notably, substantial genetic diversity was observed among the *B. garinii* sequences. Two samples were identified as *B. afzelii*, showing 98% similarity to a strain detected in *Haemaphysalis flava* from Korea (GenBank accession no. MT225121.1) (Figure 4). For relapsing fever spirochetes, a single sample was positive for *Borrelia miyamotoi*, with the 16S rRNA sequence showing 100% identity to a Russian strain (GenBank accession no. CP024351.2) (Figure 5).

### 3.3. Prevalence of Tick-Borne Pathogens

In total, seven pathogens were detected among the 232 fed ticks, with at least one pathogen present in 115 ticks (49.6%; 95% CI: 43.1–56.1%). The infection prevalence by tick species was 52.5% (107/204) in *I. persulcatus*, 43.8% (7/16) in *D. silvarum*, and 25.0% (1/4) in *H. japonica*. No pathogens were detected in *H. concinna*. *I. persulcatus* carried all seven pathogens for the tested panel, whereas *D. silvarum* and *H. japonica* harbored three and one pathogens, respectively. The prevalence of pathogens differed significantly across tick species (χ^2^ = 9.7, df = 3, *p* < 0.05). *E. muris* was detected in three tick species, with infection rates of 9.8% (20/204) in *I. persulcatus*, 12.5% (2/16) in *D. silvarum*, and 25.0% (1/4) in *H. japonica*. Both *R. conorii* subsp. *raoultii* and *B. garinii* were found in *I. persulcatus* (0.5% and 18.6%, respectively) and *D. silvarum* (25.0% and 12.5%, respectively). The remaining four pathogens were detected exclusively in *I. persulcatus* (Table 1).

### 3.4. Coinfection of Tick-Borne Pathogens

Among the 232 ticks examined, 32 were infected with two or more pathogens, yielding an overall coinfection rate of 13.8% (32/232; 95% CI: 9.9–18.8%). Dual infections were detected in 24 ticks (24/232, 10.3%). The most common coinfection was *Candidatus* R. tarasevichiae and *B. garinii* (12/232, 5.9%), followed by *Candidatus* R. tarasevichiae and *E. muris* (8/232, 3.6%), *B. garinii* and *E. muris* (2/232, 0.9%), and single cases involving *Candidatus* R. tarasevichiae with *B. afzelii* (1/232, 0.4%) and *Candidatus* R. tarasevichiae with *B. miyamotoi* (1/232, 0.4%). Triple infections were identified in 8 ticks (3.4%). The predominant pattern was *Candidatus* R. tarasevichiae, *E. muris* and *B. garinii* (6/232, 2.6%), while two additional patterns were each observed in one tick (1/232, 0.4%). One was *Candidatus* R. tarasevichiae, *E. muris*, and *B. afzelii*, and the other was *E. muris*, *B. garinii*, and *N. mikurensis*. Notably, all coinfections were detected exclusively in *I. persulcatus*, with the exception of the *B. garinii* and *E. muris* combination, which was observed in both *I. persulcatus* and *D. silvarum* (Table 2).

## 4. Discussion

This study reports the bacterial pathogens carried by ticks collected from individuals at a sentinel hospital in Northeast China. All collected ticks were semi-engorged females, a finding consistent with tick feeding ecology. Female ticks undergo a prolonged (3–7 day) engorgement phase for egg development, during which they are most likely to be noticed and removed. In contrast, male ticks feed only briefly and detach more readily, making them far less likely to be recovered in a clinical setting [22,23]. *I. persulcatus* was the predominant species, constituting 87.9% of all specimens. As a widely distributed species, *I. persulcatus* is recognized as the principal vector of human tick-borne diseases in northern China. Molecular detection identified seven pathogens, including two *Rickettsia* spp., *E. muris*, *N. mikurensis*, two *Borrelia burgdorferi* sensu lato genotypes, and *B. miyamotoi*, with an overall prevalence of 49.6%. All detected pathogens are known zoonotic agents. Notably, all pathogens were present in *I. persulcatus*, with relatively high positivity rates, and coinfections involving multiple pathogens were also observed. These findings highlight the urgent need for continuous surveillance of ticks, particularly *I. persulcatus*, and tick-borne pathogens in Northeast China, in order to better assess and mitigate the potential risks of human infection.

The most frequently detected pathogens in this study were *Rickettsia* spp. (87/232, 37.5%). Molecular analyses identified *Candidatus* R. tarasevichiae and *R. conorii* subsp. *raoultii*, both of which are established human pathogens [24]. Cases of rickettsioses have been increasing, and while many present with nonspecific or subclinical symptoms, they still carry the potential for severe outcomes. *Candidatus* R. tarasevichiae was first identified in *I. persulcatus* in Russia in 2003, and subsequently, dozens of human cases have been reported in Heilongjiang and Henan Provinces, China, with clinical manifestations including fever, headache, nausea, eschar, and lymphadenopathy [7,25]. *R. conorii* subsp. *raoultii* is associated with tick-borne lymphadenopathy (TIBOLA), characterized by necrotic erythema, eschar, and cervical lymphadenopathy [26,27]. The first human case in Northeast China was reported in 2014 [9].

The second most common pathogen detected was *B. burgdorferi* s.l., a genetically diverse complex with a broad host range and geographic distribution, and the causative agent of Lyme borreliosis involving multiple organ systems [28]. In our study, prevalence among human-biting ticks varied considerably, with *B. garinii* being the predominant genotype. *B. garinii* is a major neurotropic strain associated with neurological Lyme disease, including meningitis and radiculitis [29]. Its infection rate in *I. persulcatus* was 18.6% (38/204), suggesting a considerable risk of neurological involvement in this region. *B. afzelii*, the second most common local genotype, is strongly associated with erythema migrans, which aids in early clinical recognition [30]. These findings indicate that the *B. burgdorferi* s.l. population in this area is genetically complex, underscoring the need for enhanced molecular typing and clinical surveillance.

*Ehrlichia muris*, the pathogen responsible for human ehrlichiosis, was also detected. In 2009, a novel pathogen closely related to *E. muris* was confirmed as the causative agent of human ehrlichiosis in the United States [31]. In this study, *E. muris* was detected in three tick species—*I. persulcatus*, *D. silvarum*, and *H. japonica*—with an overall prevalence of 9.9% (23/232). Although no human cases of *E. muris* infection have been reported in China to date, our findings suggest a potential risk of ehrlichiosis in this region.

Importantly, a substantial proportion of positive ticks carried multiple pathogens, with coinfections observed in 32 of 115 infected ticks (27.8%). Among these, the majority (12/32, 37.5%) involved coinfections of *Candidatus* R. tarasevichiae. and *B. garinii*, primarily in *I. persulcatus*. Frequent coinfections in *I. persulcatus* may be attributable to its broad host range, encompassing multiple vertebrate species that harbor diverse pathogens. Such coinfections may result from simultaneous transmission during blood feeding or sequential acquisition from multiple hosts [32]. The presence of multiple pathogens in ticks highlights the risk of human and animal coinfections, which should be considered in the diagnosis and treatment of tick-borne diseases. In endemic areas, humans may be exposed to more than one pathogen concurrently, potentially leading to more severe disease outcomes or atypical clinical manifestations, thereby complicating diagnosis and management [33].

Laboratory evidence confirms that the principal tick species identified in our study possess vector competence for transmitting the respective pathogens. For *I. persulcatus*, experimental studies have demonstrated its ability to transmit *Candidatus* R. tarasevichiae and *E. muris* both transstadially and transovarially, and to infect murine models via infectious saliva within 24 h of attachment [34]. *R. conorii* subsp. *raoultii* has been maintained with high transovarial efficiency across four generations in *D. silvarum*, and the competence of this tick species to transmit the pathogen to murine hosts has been confirmed experimentally [35]. Furthermore, studies have also reported that *I. persulcatus* can carry up to 51 microbial agents, including *Candidatus* R. tarasevichiae, *R. conorii* subsp. *raoultii*, *Anaplasma phagocytophilum*, *Candidatus* N. mikurensis, *B. afzelii*, *B. miyamotoi*, *B. garinii* and *E. muris*, demonstrating the remarkable pathogen-carrying capacity of this tick species [36]. Further literature indicates that *D. silvarum* can serve as an efficient vector for a variety of tick-borne pathogens, including *R. slovaca*, *R. conorii* subsp. *raoultii*, *A. phagocytophilum*, among others. Notably, *R. conorii* subsp. *raoultii* has been identified as the predominant *Rickettsia* species in *D. silvarum*, reinforcing the vector potential of this tick species in pathogen transmission cycles [16,37]. These findings fulfill the requirements for both pathogen maintenance across life stages and transmission between hosts, confirming that the PCR-positive signals observed in our study represent active vector–pathogen associations rather than recent feeding contamination.

Beyond cataloguing pathogens, the findings also reflect the tick’s life-history strategy. Specifically, the engorged female ticks align with their prolonged attachment phase, during which salivary secretion of pathogens is maximized [38]. Within this prolonged meal, the mid-gut lumen becomes heme-rich yet reactive-oxygen-species-buffered milieu that activates the *Rickettsia* type-IV secretion system and sustains transstadial survival of spirochetes for up to ten months—thereby establishing the physiological foundation for effective vector competence [23,29]. Consequently, the high pathogen prevalence reported here does not represent passive contamination but rather a predictable outcome of tick-specific adaptations—including feeding physiology, antioxidant homeostasis, and host-seeking behavior—that collectively enable persistent pathogen carriage and long-distance dispersal.

This study has several limitations. First, all tick samples were collected from patients who actively sought medical care with attached ticks. In these cases, the duration of tick attachment was generally short, and most patients had not yet developed clinical symptoms. Moreover, many received prophylactic treatment following tick removal, which limited our ability to assess the clinical progression of infections. Second, as this study focused specifically on pathogen identification in ticks, no individual epidemiological data were collected from the participants, no follow-up was conducted, preventing long-term evaluation of disease incidence, progression, or treatment outcomes.

Despite these limitations, our findings provide evidence that bacterial tick-borne pathogens pose a significant public health threat in Northeast China and offer important insights to guide the early prevention and targeted management of tick-borne diseases in the region.

## 5. Conclusions

This study detected seven zoonotic bacterial pathogens in 232 human-derived fed ticks from a sentinel hospital in Northeast China (May–August 2023–2024), with an overall pathogen positivity rate of 49.6% (95% CI: 43.1–56.1%) and a coinfection rate of 13.8% (95% CI: 9.9–18.8%). *I. persulcatus* (87.9% of total ticks) was the dominant species and the sole carrier of all seven pathogens. These results confirm a substantial public health threat of tick-borne bacterial diseases in Northeast China, emphasizing the urgent need for continuous surveillance of *I. persulcatus* and its associated pathogens, as well as the implementation of targeted tick-bite prevention strategies to reduce human infection risks.

## Figures and Tables

**Table 1:** Prevalence of various pathogens in ticks from Mudanjiang Forestry Central Hospital in China, 2023–2024.

Pathogens	No. of Positive (%, 95%CI)				
	*I. persulcatus* (n = 204)	*D. silvarum* (n = 16)	*H. concinna* (n = 8)	*H. japonica* (n = 4)	Total (n = 232)
*Ca*. R. tarasevichiae	82 (40.2, 33.7–47.1)	0 (0)	0 (0)	0 (0)	82 (35.3, 29.5–41.7)
*R. conorii* subsp. *raoultii*	1 (0.5, 0.1–2.7)	4 (25.0, 10.2–49.5)	0 (0)	0 (0)	5 (2.2, 0.9–4.9)
*E. muris*	20 (9.8, 6.4–14.7)	2 (12.5, 3.5–36.0)	0 (0)	1 (25.0, 4.56–69.9)	23 (9.9, 6.7–14.4)
*N.mikurensis*	1 (0.5, 0.1–2.7)	0 (0)	0 (0)	0 (0)	1 (0.4, 0.1–2.4)
*B. garinii*	38 (18.6, 13.9–24.5)	2 (12.5, 3.5–36.0)	0 (0)	0 (0)	40 (17.2, 12.9–22.6)
*B. afzelii*	2 (1, 0.3–3.5)	0 (0)	0 (0)	0 (0)	2 (0.9, 0.2–3.1)
*B. miyamotoi*	1 (0.5, 0.1–2.7)	0 (0)	0 (0)	0 (0)	1 (0.4, 0.1–2.4)
Overall	107 (52.5, 45.3–59.5)	7 (43.8, 21.1–68.6)	0 (0)	1 (25.0, 1.3–73.8)	115 (49.6, 43.1–56.1)

**Table 2:** Pathogens coinfection in ticks from Mudanjiang Forestry Central Hospital in China, 2023–2024.

Pathogens Coinfection	No. of Positive (%, 95%CI)				
	*I. persulcatus* (n = 204)	*D. silvarum* (n = 16)	*H. concinna* (n = 8)	*H. japonica* (n = 4)	Total (n = 232)
*Ca.* R. tarasevichiae + *E. muris*	8 (3.92, 2~7.55)	0 (0)	0 (0)	0 (0)	8 (3.45, 1.76~6.66)
*Ca.* R. tarasevichiae + *B. garinii*	12 (5.88, 3.4~10)	0 (0)	0 (0)	0 (0)	12 (5.17, 2.98~8.82)
*Ca.* R. tarasevichiae + *B. afzelii*	1 (0.49, 0.09~2.72)	0 (0)	0 (0)	0 (0)	1 (0.43, 0.08~2.4)
*Ca.* R. tarasevichiae + *B. miyamotoi*	1 (0.49, 0.09~2.72)	0 (0)	0 (0)	0 (0)	1 (0.43, 0.08~2.4)
*B. garinii + E. muris*	1 (0.49, 0.09~2.72)	1 (6.25, 1.11~28.33)	0 (0)	0 (0)	2 (0.86, 0.24~3.09)
*Ca.* R. tarasevichiae + *E. muris* + *B. garinii*	6 (2.94, 1.35~6.27)	0 (0)	0 (0)	0 (0)	6 (2.59, 1.19~5.53)
*Ca.* R. tarasevichiae + *E. muris* + *B.afzelii*	1 (0.49, 0.09~2.72)	0 (0)	0 (0)	0 (0)	1 (0.43, 0.08~2.4)
*E. muris* + *B. garinii* + *N. mikurensis*	1 (0.49, 0.09~2.72)	0 (0)	0 (0)	0 (0)	1 (0.43, 0.08~2.4)
Total	31 (15.2, 10.92~20.76)	1 (6.25, 1.11~28.33)	0 (0)	0 (0)	32 (13.79, 9.94~18.82)

## Data Availability

All datasets supporting the conclusions of this article are included within the article and its Supplementary Materials. All accession numbers corresponding to the positive sequences detected in this study are listed in Table S2. The data of reference sequences involved in this study are openly available in GenBank (https://www.ncbi.nlm.nih.gov/genbank/).

## Supplementary Materials

The following supporting information can be downloaded at: https://www.mdpi.com/article/10.3390/microorganisms13122862/s1, Table S1: Sequence, annealing temperature and amplicon size of primers used for detecting in this study; Table S2: GenBank accession numbers of pathogens deposited in GenBank. References [9,10,39,40,41,42,43] are cited in the Supplementary Materials.

## Abbreviations

The following abbreviations are used in this manuscript:

PCR	Polymerase Chain Reaction
ML	Maximum Likelihood
CI	Confidence Interval
s.l.	sensu lato
