# Supplementary material for: Variety of Bacterial Pathogens in Ticks Removed from Humans, Northeastern China
Source: Microorganisms. 2025 Dec 16;13(12):2862. doi: 10.3390/microorganisms13122862 (PMC12736349; doi:10.3390/microorganisms13122862)
Supplement: Supplementary file 1 [file microorganisms-13-02862-s001.zip › Table S1.pdf]

Table S1. Sequence, annealing temperature and amplicon size of primers used for detecting in this study

Pathogens	Gene	Primer	Sequence [5'→3']	Annealing temperature [℃]	Amplicon size [bp]	Note	Reference
Rickettsia spp.	*gltA*	CS2d	ATGACCAATGAAAATAATAAT	50	1100	first–PCR	[9]
		CSEndr	CTTATACTCTCTATGTACA				
		RpCS877f	GGGGACCTGCTCACGGCGG	52	381	nested–PCR	
		RpCS1258r	ATTGCAAAAAGTACAGTGAACA				
	ompA	70F	ATGGCGAATATTTCTCCAAAA	50	631	first–PCR	[9]
		701R	GTTCCGTTAATGGCAGCATCT				
		70F	ATGGCGAATATTTCTCCAAAA	50	532	semi nested–PCR	
		602R	AGTGCAGCATTCGCTCCCCCT				
	17 kDa	17 k3	GCTTTACAAAATTCTAAAAACCATATA	52	547	first–PCR	[39]
		17 k5	TGTCTATCAATTCACAACTTGCC				
		Tara17KD13s1	ATTGTCCGTCAGGTTGGC	52	395	nested–PCR	
		Tara17KD408r1	CGGGCGGTATGAATAAGC				
Ehrlichia spp.	16S rRNA	EHR1-out	GAACGAACGCTGGCGGCAAGC	60	691	first–PCR	[40]
		EHR2-out	AGTA[T/C]CG[A/G]ACCAGATAGCCGC				
		EHR3-in	TGCATAGGAATCTACCTAGTAG	52	524	nested–PCR	
		EHR4-in	CTAGGAATTCCGCTATCCTCT				
	gltA	Eh-gltA-112F1	GGRRTRTTAACTTATGATCCAGG	55	575	first–PCR	[41]
		Eh-gltA-686R1	GCATTYTGYTCATGATCAGCATG				
		Eh-gltA-137F2	TTATGTCTACTCGCTGCTTGTGA	55	478	nested–PCR	
		Eh-gltA-614R2	TARGAAGAAAYRTCAAACATCATATG				
	groEL	gro607F	GAAGATGCWGTWGGWTGTACKGC	*	730	first–PCR	This study
		gro1294R	AGMGCTTCWCCTTCWACRTCYTC				
		gro677F	ATTACTCAGAGTGCTTCTCAATG	†	364	nested–PCR	
		gro1121R	TGCATACCATCAGTTTTTTCAAC				
Borrelia spp.	5S-23S rRNA	23S3	CGACCTTCTTCGCCTTAAAGC	55	412	first–PCR	[42]
		23Sa	TAAGCTGACTAATACTAATTACCC				
		23S5	CTGCGAGTTCGCGGGAGA	59	253	nested–PCR	
		23S6	TCCTAGGCATTCACCATA				
	16S rRNA	Brm1	CGCTGTAAACGATGCACACTTGG TGTTAATC	60	500	first–PCR	[43]
		Brm2	CGGCAGTCTCGTCTGAGTCCCCATCT				
Neoehrlichia mikurensis	16S rRNA	Eh-out1	TTGAGAGTTTGATCCTGGCTCAGAACG	50	1501	first–PCR	[10]
		3–17U	WAAGGWGGTAATCCAGC				
		EHR16SD	GGTACCYACAGAAGAAGTCC	52	312	nested–PCR	
		EHR16SR	TAGCACTCATCGTTTACAGC				
	groEL	gro607F	GAAGATGCWGTWGGWTGTACKGC	*	730	first–PCR	[41]
		gro1294R	AGMGCTTCWCCTTCWACRTCYTC				
		gro677F	ATTACTCAGAGTGCTTCTCARTG	†	364	nested–PCR	
		gro1121R	TGCATACCRTCAGTYTTTTCAAC				

*This step was set as 10 cycles of 58°C for 30s and 72°C for 45s, followed by 25 cycles of 53°C for 30s and 72°C for 45s.

†This step was set as 10 cycles of 56°C for 30s and 72°C for 35s, followed by 25 cycles of 53°C for 30s and 72°C for 35s.
