# Supplementary material for: Variety of Bacterial Pathogens in Ticks Removed from Humans, Northeastern China
Source: Microorganisms. 2025 Dec 16;13(12):2862. doi: 10.3390/microorganisms13122862 (PMC12736349; doi:10.3390/microorganisms13122862)
Supplement: Supplementary file 1 [file microorganisms-13-02862-s001.zip › Table S2.pdf]

Table S2. GenBank accession numbers of pathogens deposited in GenBank

Pathogens	Gene	GenBank accession numbers
Rickettsia conorii subsp. *raoultii*	*ompA*	PV521702-PV521706
	gltA	PV528074;<br>PV528076-PV528079
	17 kDa	PV478074-PV478078
Candidatus Rickettsia tarasevichiae	*ompA*	PV521710-PV521719;<br>PV521721-PV521753;<br>PV521755-PV521756;<br>PV521758-PV521780;<br>PV521782-PV521792;<br>PV521794-PV521796
	gltA	PV528081-PV528085;<br>PV528087-PV528091;<br>PV528093-PV528098;<br>PV528100-PV528106;<br>PV528109-PV528113;<br>PV528115-PV528116;<br>PV528118-PV528122;<br>PV528124-PV528131;<br>PV528133;<br>PV528135;<br>PV528137-PV528140;<br>PV528142-PV528145;<br>PV528147-PV528148;<br>PV528151-PV528154;<br>PV528156-PV528157;<br>PV528159-PV528162;<br>PV528164;<br>PV528166-PV528171;<br>PV528173-PV528174;<br>PV528176-PV528181;<br>PV528185-PV528186
	17 kDa	PV521601-PV521607;<br>PV521609-PV521627;<br>PV521630-PV521647;<br>PV521650-PV521655;<br>PV521657-PV521661;<br>PV521663;<br>PV521665-PV521667;<br>PV521670-PV521671;<br>PV521673-PV521675;<br>PV521677-PV521679;<br>PV521681-PV521688;<br>PV521690-PV521691;<br>PV521694;<br>PV521696-PV521699
Ehrlichia muris	16S rRNA	PV505460-PV505462;<br>PV505464-PV505469;<br>PV505471-PV505481;<br>PV505483-PV505485
	gltA	PV528187-PV528189;<br>PV528191-PV528196;<br>PV528197-PV528203;<br>PV528205-PV528208;<br>PV528210-PV528212
	groEL	PV528213-PV528234;<br>PX233349
Borreliella afzelii	5S-23S rRNA	PV557988-PV557989
Borreliella garinii	5S-23S rRNA	PV557990-PV558029

Borrelia miyamotoi	16S rRNA	PV562577
Neoehrlichia mikurensis	16S rRNA	PV562797
	groEL	PV528235
